# Association of mitochondrial RNA expression levels in saliva and plasma with interferon signature gene expression and disease activity in patients with Sjögren disease

**DOI:** 10.1136/rmdopen-2024-005166

**Published:** 2025-05-13

**Authors:** You-Jung Ha, Yong Seok Choi, Se Rim Choi, Jimin Yoon, Doyeong Ku, Yoosik Kim, Eun Ha Kang, Keun-Suh Kim, Woo-Jin Jeong, Joon Young Hyon, Seunghee Cha, Yun Jong Lee

**Affiliations:** 1 Division of Rheumatology, Department of Internal Medicine, Seoul National University Bundang Hospital, Seongnam, Gyeonggi, Korea (the Republic of); 2 Department of Internal Medicine, Seoul National University College of Medicine, Seoul, Korea (the Republic of); 3 Medical Science Research Institute, Seoul National University Bundang Hospital, Seongnam, Gyeonggi, Korea (the Republic of); 4 Department of Chemical and Biomolecular Engineering, Korea Advanced Institute of Science and Technology, Daejeon, Korea (the Republic of); 5 Department of Periodontology, Section of Dentistry, Seoul National University Bundang Hospital, Seongnam, Gyeonggi, Korea (the Republic of); 6 Department of Otorhinolaryngology - Head & Neck Surgery, Seoul National University Bundang Hospital, Seongnam, Gyeonggi, Korea (the Republic of); 7 Department of Ophthalmology, Seoul National University Bundang Hospital, Seongnam, Gyeonggi, Korea (the Republic of); 8 Division of Oral Medicine, Department of Oral and Maxillofacial Diagnostic Sciences, University of Florida, Gainesville, Florida, USA; 9 Department of Medical Device Development, Seoul National University College of Medicine, Seoul, Republic of Korea

**Keywords:** Sjogren's Syndrome, Biomarkers, Autoimmune Diseases

## Abstract

**Objective:**

To unveil the clinical implications of mitochondrial RNAs (mt-RNAs) in Sjögren disease (SjD), this study evaluated mt-RNA expression levels in the plasma and saliva of patients with SS and their association with SjD-related features.

**Methods:**

Plasma, saliva and/or peripheral blood mononuclear cells (PBMCs) were collected from 111 patients with SjD and 35 healthy controls (HCs), with 40 rheumatoid arthritis (RA) and 40 systemic lupus erythematosus (SLE) disease controls. The expression levels of mt-RNAs and interferon-stimulated genes (ISGs) were quantified by real-time PCR. Composite mt-RNA and ISG scores were calculated using logistic regression models. Their discriminative power was evaluated using receiver operating characteristic curve analyses, and correlations with clinical data were explored.

**Results:**

Altered mt-RNA expression in saliva or plasma and ISG expression in PBMCs were detected in patients with SjD, compared with HCs. Saliva and plasma mt-RNA scores showed better discriminative ability (area under the curve values=0.847 and 0.789, respectively) than ISG scores in distinguishing SjD from HCs. Plasma mt-RNA scores were significantly higher in patients with SjD than in those with RA and SLE (p<0.05). Saliva mt-RNA scores were positively associated with objective disease activity measures and Raynaud phenomenon in patients with SjD, whereas plasma mt-RNA scores did not show this association. RA and SLE disease activity correlated with plasma mt-RNA scores.

**Conclusions:**

Extracellular mt-RNA burden is elevated in SjD, and mt-RNA scores effectively discriminated patients with SjD from HCs. Saliva mt-RNA levels were associated with SjD disease activity, suggesting their potential utility in disease monitoring and stratification of SjD.

**WHAT IS ALREADY KNOWN ON THIS TOPIC**

**WHAT THIS STUDY ADDS**

- We identified an increased extracellular mt-RNA burden in the saliva and plasma of patients with SjD compared with healthy controls, with higher plasma mt-RNA scores than those in patients with rheumatoid arthritis and systemic lupus erythematosus.
- Significant associations were found between salivary mt-RNA levels and the European Alliance of Associations for Rheumatology Sjögren’s syndrome disease activity index as well as the presence of Raynaud phenomenon in SjD.

**HOW THIS STUDY MIGHT AFFECT RESEARCH, PRACTICE OR POLICY**

- Plasma and salivary mt-RNA levels may serve as novel potential biomarkers for disease activity and stratification of SjD.

## Introduction

 Sjögren disease (SjD) is a systemic autoimmune disease that primarily affects the salivary and lacrimal glands, leading to dryness of the mouth and eyes.^1^ The main immunohistopathological feature is focal periepithelial mononuclear cell infiltration, including dendritic cells (DCs), CD4+T cells, B cells and plasma cells in the exocrine glands. Although the initial organ involved in SjD is unknown, the disease is characterised by epithelial cell damage with subsequent autoantigen release and is sometimes referred to as ‘autoimmune epithelitis’.^2^

Following epithelial cell damage in SjD, the innate immune response is triggered by the stimulation of pattern recognition receptors (PRRs) and the activation of DCs and macrophages. This leads to the release of interferons (IFNs), activation of CD4+ cells and induction of B cell stimulatory cytokines, such as the B cell activating factor of the tumour necrosis factor (TNF) family and a proliferation-inducing ligand. Then, activated B cells drive autoantibody production, forming immune complexes that perpetuate overactivation of the immune response.^1^

Although environmental factors, including viruses, may influence these pathological processes, there is insufficient evidence to confirm that viruses are the key initiating factors in SjD pathogenesis.^2^ Growing evidence has suggested that nucleic acids (NAs) can activate the innate immune response even in the absence of external stimuli, for example, viral infection,^3^ and may serve as potential triggers for the type I IFN system in autoimmune diseases.^4^

Immunostimulatory self-DNA may originate from endogenous retroelements, leaked mitochondrial DNA (mt-DNA) or uptake of extracellular DNA following tissue damage.^5^ Additionally, proinflammatory RNAs may be derived from endogenous retroelements, mis-edited or altered processed RNAs, or mt-RNA.^6^ Endogenous DNAs/RNAs induce an innate immune response through various NA sensors, including cyclic GMP-AMP synthase (cGAS)/stimulator of interferon genes, retinoic acid–inducible gene I (RIG I)-like receptors, RNA-activated protein kinase (PKR), and Toll-like receptors (TLRs).^5 6^ Consequently, it has been hypothesised that an inappropriate increase in immunostimulatory endogenous NAs in genetically susceptible individuals could chronically activate the type I IFN system in patients with SjD.

mt-RNAs are transcribed from the circular mitochondrial genome containing two strands: heavy and light. They form intermolecular double-stranded RNAs (dsRNAs), which can also be recognised by PRRs such as TLRs or RIG-1-like receptors.^7^ Recently, we reported that mt-RNAs participate in type I IFN induction and salivary epithelial dysfunction.^8^ In that study, the levels of saliva and tear mt-RNA were significantly elevated in patients with SjD compared with non-SjD sicca patients or healthy controls (HCs). However, no studies have investigated mt-RNA expression in plasma samples or the clinical implications of mt-RNA levels in patients with SjD or disease control of rheumatoid arthritis (RA) or systemic lupus erythematosus (SLE).

Therefore, this study aimed to determine the clinical importance of mt-RNAs in SjD by measuring mt-RNA and IFN-stimulated gene (ISG) expressions in patient samples, comparing them with control groups and correlating these findings with clinical parameters.

## Methods

### Study population and sample collection

Between September 2010 and August 2023, 147 individuals were consecutively enrolled in two SjD studies (IRB numbers (nos.): B0506-021-004 and B-2111-722-301) at our institute. Of these, 125 donated at least two types of samples (plasma, saliva or peripheral blood mononuclear cells (PBMCs)); however, 14 were excluded because of insufficient specimen volume, resulting in 111 patients included in the analysis. All included patients met the 2016 American College of Rheumatology (ACR)/European Alliance of Associations for Rheumatology (EULAR) classification criteria for Sjögren syndrome.^9^ At enrolment, blood was drawn using the Vacutainer CPT mononuclear cell preparation tube with sodium citrate (BD Biosciences, San Jose, California, USA), and plasma and isolated PBMC samples were obtained. Unstimulated whole saliva (UWS) was collected via passive drooling. As a control group, we recruited 35 age-matched HCs and collected their plasma and UWS samples. Plasma samples from 40 age-matched patients with RA or SLE, who were enrolled in other studies (IRB nos.: B-0905-075-013 and B-2209-778-304), were used as autoimmune disease controls. Patients with SLE met the 2012 ACR/SLICC criteria or the 2019 ACR/EULAR criteria for the classification of SLE,^10 11^ and patients with RA met the 2010 ACR/EULAR classification criteria.^12^ Characteristics of the patients and controls and the number of samples obtained are summarised in online supplemental table 1. Collected plasma and UWS samples were stored at −80°C until analysis.

### Collection of clinical data and definition of variables

We collected data on patient demographics, disease duration, clinical manifestations and laboratory findings on SjD diagnosis and comorbidities. Laboratory findings included complete blood count; erythrocyte sedimentation rate (ESR); complements C3 and C4; autoantibodies, such as rheumatoid factor, antinuclear antibody, anti-SSA/Ro, anti-SSB/La, and anticentromere antibody; immunoglobulin G; β2-microglobulin and cryoglobulin. Lymphopenia was defined as an absolute lymphocyte count <1000/mm^3^. For SjD-related clinical assessments, we collected the EULAR Sjögren’s Syndrome Disease Activity Index (ESSDAI), clinical ESSDAI (ClinESSDAI), Patient Reported Index (ESSPRI) and EuroQol 5 Dimension (EQ-5D) data at enrolment.^13^,^16^ Histopathological assessment for the inflammation severity of minor salivary gland was performed using modified semiquantitative focus score (FS; categorised as 0, <1, ≥1 and <2, and ≥2).^17^ For RA and SLE, we calculated disease activity indices, for example, the Disease Activity Score-28 with ESR (DAS28) and SLE Disease Activity Index-2000 (SLEDAI) at the time of sample collection.

### Sample processing and measurement of mt-RNA expression

PBMCs were isolated using the CPT mononuclear tube via centrifugation. Total RNA was extracted from cryopreserved PBMCs using the RNeasy Micro Kit (Qiagen, Redwood City, California, USA). Additionally, total RNA was extracted from 200 µL of citrate plasma using the miRNeasy Serum/Plasma Advanced kit (Qiagen) and from 50 to 200 µL of UWS using the RNeasy Protect Saliva Mini Kit (Qiagen). Subsequently, a reverse transcription (RT) reaction was performed to synthesise the first-strand complementary DNA (cDNA) using SuperScript IV RTase (Invitrogen, Carlsbad, California, USA). The synthesised cDNA was subjected to quantitative real-time PCR (qRT-PCR) in a 20 µL reaction volume containing SensiFAST SYBR Lo-ROX reagent (Bioline, Taunton, Massachusetts, USA). RT-quantitative PCR was performed using the ABI 7500 RT-PCR System (Applied Biosystems, Foster City, California, USA).

We evaluated the expressions of seven mt-RNA genes in the plasma and saliva samples: *CO1* (heavy strand), *CO2* (heavy strand), *ND1* (light strand), *ND4* (heavy strand), *ND5* (heavy strand), *ND6* (light strand) and *CYTB* (heavy strand). This selection was based on the results of our previous study that demonstrated the top five differentially expressed mt-RNAs in the tears and saliva of patients with SjD (*CYTB, ND4, ND1, CO1* and *CO2* in tear, and *ND5, CO1, ND1, ND6* and *CYTB* in saliva).^8^ To estimate the ISG signature from PBMCs, we assessed the expression levels of eight transcripts: IFN-induced protein with tetratricopeptide repeats 1 (*IFIT1*), *IFIT3*, IFN-induced protein 44 (*IFI44*), IFI44-like (*IFI44L*), lymphocyte antigen 6 complex, locus E (*LY6E*), 2-5 oligoadenylate synthetase 1 (*OAS1*), myxovirus resistance 1 (*MX1*) and ISG15 ubiquitin-like modifier (*ISG15*), on the basis of previous studies.^18^,^20^ The primers used are listed in online supplemental table 2.

The relative gene expression of each mt-RNA and ISG was calculated using the z-score method, with normalisation based on the levels of HCs as follows^18^:


$$z-score\;for\;each\;gene=\frac{individual\;target\;gene\;expression-mean\;\left(HC\;target\;gene\;expression\right)}{standard\;deviation\;\left(HC\;target\;gene\;expression\right)}$$

### Statistical analyses

Comparisons between the two groups were conducted using a *t*-test for continuous variables and χ^2^ or Fisher’s exact test for categorical variables. The Pearson’s correlation was used to analyse the correlation between two continuous variables. Analysis of variance with the Tukey post hoc test was used to compare means between more than two groups. Univariate regression analyses were used to identify mt-RNAs or ISGs significantly associated with SjD. Variables with a p<0.1 in the univariable analyses were included in sex-adjusted and age-adjusted multivariate logistic regression analyses using a forward selection method. A composite score for mt-RNA (mt-RNA score) or ISG (ISG score) expression was created on the basis of regression coefficients from multivariate logistic regression analysis. To assess the diagnostic performance of mt-RNA scores, receiver operating characteristic (ROC) curve analysis and area under the curve (AUC) estimation were performed to determine the ability of mt-RNA scores and ISG score to discriminate SjD. DeLong’s test was used to compare ROC curves. Since only 26 SjD patients had all three kinds of samples, we used the KNN algorithm to impute missing values and performed 1000 bootstrap replications for internal validation, given the lack of an external dataset.^21^ Additionally, post hoc sensitivity analyses were performed with patients stratified based on anti-SSA/Ro positivity, FS≥1 or serum IgG ≥1.8 g/dL. All statistical analyses were performed using SPSS Statistics for Windows (V.25; IBM) and R (V.4.4.1), and a p<0.05 was considered statistically significant.

## Results

### Baseline characteristics of the study population

The baseline characteristics of the patients with SjD are summarised in table 1. The average age at enrolment was 47.8 years, and 106 patients (95.5%) were female. Plasma, UWS and PBMC samples were analysed from 79 (71.2%), 65 (58.6%) and 103 (92.8%) patients, respectively. The mean ESSDAI, ClinESSDAI and ESSPRI scores were 3.7, 2.6, and 4.9, respectively. 25 patients (22.5%) had moderate-to-high disease activity (ESSDAI≥5), and 60 out of 102 patients (58.8%) had unsatisfactory symptom states (ESSPRI≥5). Pilocarpine was prescribed to 19 (17.1%) patients with SjD. Additionally, 25 (22.5%) patients with SjD regularly took immunoregulatory drugs, including hydroxychloroquine (n=22), glucocorticoids (n=9), methotrexate (n=1), mycophenolate (n=1) and azathioprine (n=1). In comparison, 18 (45.0%) patients with RA and 35 (87.5%) patients with SLE received immunoregulatory agents (online supplemental table 3).

**Table 1:** Baseline characteristics of the included patients with SjD (n=111)

Characteristics	
Age (year)	47.8±11.8
Women	106 (95.5)
Disease duration (year) after SjD diagnosis	1.0±2.0
Menopause	48/106 (45.3)
Smoking history	
Current smoker	2 (1.8)
Ever smoker	20 (18.0)
Comorbidities	
Hypothyroidism	16 (14.4)
Primary biliary cirrhosis	3 (2.7)
Autoimmune hepatitis	1 (0.9)
Hypertension	11 (14.4)
Diabetes mellitus	1 (0.9)
Clinical features at SjD diagnosis	
Oral dryness	71 (64.0)
Ocular dryness	80 (72.1)
Salivary gland dysfunction*	87/108 (80.6)
UWSF rate (mL/min)	0.08±0.10
SWSF rate (mL/min)	0.47±0.45
Lacrimal gland dysfunction†	91/109 (82.0)
Schirmer test ≤5/5 mm	79/100 (79.0)
Ocular staining score ≥5	73/106 (68.9)
Anti-SSA/Ro positivity	101 (91.0)
Anti-SSB/La positivity	61 (55.0)
Focal lymphocytic sialadenitis with focus score ≥1	77/101 (76.2)
Extra-glandular manifestations	
Arthralgia	32 (28.8)
Raynaud phenomenon	18 (16.2)
Skin rashes	5 (4.5)
Peripheral neuropathy	2 (1.8)
Renal tubular acidosis	2 (1.8)
Interstitial lung disease	5 (4.5)
Disease assessments at enrollment	
ESSDAI	3.7±3.1
ESSDAI≥5	25 (22.5)
ClinESSDAI	2.6±3.0
ClinESSDAI≥5	19 (17.1)
ESSPRI	4.9±2.1
Dryness VAS	5.7±0.2
Limb pain VAS	3.4±0.3
Fatigue VAS	5.6±0.3
ESSPRI≥5	60/102 (58.8)
EQ-5D	0.832±0.120
Laboratory findings at enrollment	
White cell count (x10^9^/L)	4.53±1.26
Lymphopenia (<1000/mm)	45 (40.5)
Hb (g/L)	127±11
Platelet (x10^9^/L)	227±56
ESR (mm/hour)	33.3±24.3
IgG (g/dL)	2023±633
β2-microglobulin (mg/L)	2.30±0.70
High β2-microglobulin (≥2 mg/L)	37/107 (33.3)
C3 (mg/dL)	98.5±16.9
C4 (mg/dL)	20.6±7.8
Hypocomplementaemia	6/110 (5.4)

Values are presented as mean±SD or number (%).

* Defined by Schirmer test ≤5 mm or ocular staining score ≥5.

† Defined by abnormal salivary gland scan findings or UWSF≤0.1 mL/min.

ClinESSDAI, clinical ESSDAI; EQ-5D, EuroQoL 5 Dimension; ESR, erythrocyte sedimentation rate; ESSDAI, EULAR Sjögren’s Syndrome Disease Activity Index; ESSPRI, EULAR Sjögren’s Syndrome Patient Reported Index; EULAR, European Alliance of Associations for Rheumatology; Hb, haemoglobin; SjD, Sjögren disease; SWSF, stimulated whole salivary flow; UWSF, unstimulated whole salivary flow; VAS, Visual Analogue Scale.

### Comparisons of mt-RNA and ISG expressions between patients with SjD and HCs

**Figure 1:**
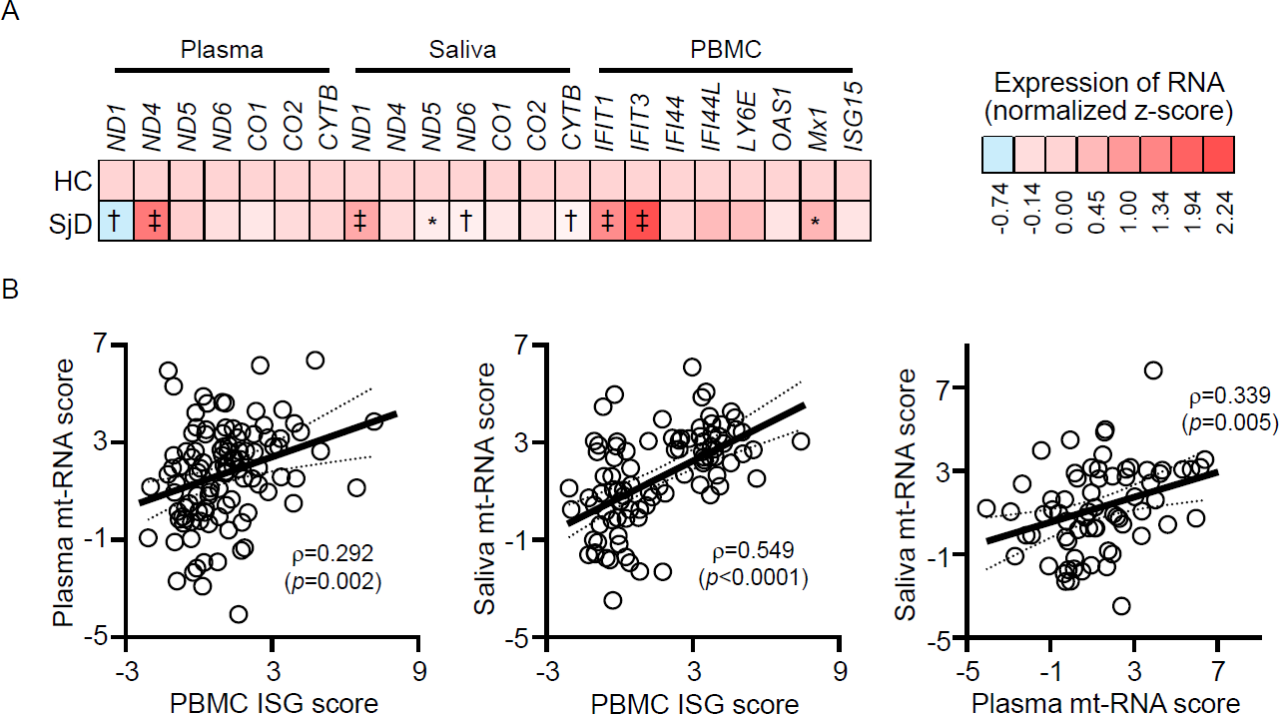
(**A**) Heatmap showing the relative expression levels of mitochondrial RNA (mt-RNA) (normalised z-scores) in the plasma and unstimulated saliva and interferon-stimulated genes (ISGs) in peripheral blood mononuclear cells (PBMCs) of patients with Sjögren disease (SjD) compared with healthy controls (HCs). (**B**) Pairwise correlations among PMBC ISG scores and plasma and salivary mt-RNA scores (ρ, Pearson correlation coefficient). *p<0.05, ^†^p<0.01, ^‡^p<0.001.

Compared with HCs, plasma *ND1* levels decreased and plasma *ND4* levels increased in patients with SjD, whereas salivary *ND1* levels increased and salivary *ND5*, *ND6* and *CYTB* levels decreased. In PBMCs, the expressions of *IFIT1*, *IFIT3* and *MX1* were significantly upregulated in patients with SjD (figure 1). Although mt-RNA levels in the salivary glands increased with age of NOD mice,^8^ saliva and plasma mt-RNA levels were not significantly correlated with age in patients with SjD, except for salivary *ND1* (*ρ*=0.254, p=0.04).

### Correlations between plasma or saliva mt-RNA and PBMCs ISG expression

We investigated the correlation between plasma mt-RNA, salivary mt-RNA and PBMC ISG expression levels using pooled data from HCs and patients with SjD. Some salivary mt-RNAs were significantly associated with PBMC ISG expression levels. Salivary *ND1* levels were significantly and positively correlated with the expressions of *IFIT1*, *IFIT3*, *IFI44L*, *LY6E* and *MX1* in PBMCs (online supplemental figure 1A). However, no significant positive correlations were found between most PBMC ISGs and plasma mt-RNA levels, or between plasma and salivary mt-RNA levels (online supplemental figure 1B, C).

### Development of mt-RNA scores and their ability to discriminate patients with SjD

We analysed mt-RNA and ISG expression data from patients with SjD and HCs using univariate and multivariate logistic regression analyses, with SjD as the dependent variable, adjusting for age and sex (table 2). Significant predictors from the multivariate analysis were used to create a composite mt-RNA and ISG scoring system. The mt-RNA score was calculated as (0.944×normalised z-score of *ND4*)–(1.543×normalised z-score of *ND1*) for plasma and (2.151×normalised z-score of *ND1*)–(1.141×normalised z-score of *ND5*)–(1.239×normalised z-score of *ND6*) for saliva. The ISG score in the PBMCs was defined as follows: (0.722×normalised z-score of *IFIT1*)+(0.741×normalised z-score of *IFIT3*)–(0.715×normalised z-score of *LY6E*). After calculating these weighted transcript burden scores, all three scores were found to be significantly correlated (all p<0.01; figure 1B).

**Table 2:** Age-adjusted and sex-adjusted logistic regression analysis of mt-RNAs or interferon-stimulated genes expression for discriminating patients with Sjögren disease from healthy controls

Variables	Univariate			Multivariate*		
	B	OR (95% CI)	P value	B	OR (95% CI)	P value
Plasma *ND1*	−0.566	0.568 (0.384 to 0.840)	0.005	−1.543	0.214 (0.109 to 0.418)	<0.001
Plasma *ND4*	0.418	1.519 (1.190 to 1.939)	0.001	0.944	2.571 (1.745 to 3.786)	<0.001
Plasma *ND5*	0.060	1.062 (0.763 to 1.478)	0.721			
Plasma *ND6*	−0.091	0.913 (0.680 to 1.225)	0.544			
Plasma *CO1*	−0.162	0.851 (0.636 to 1.138)	0.276			
Plasma *CO2*	−0.057	0.944 (0.693 to 1.287)	0.716			
Plasma *CYTB*	−0.003	0.997 (0.674 to 1.475)	0.986			
Saliva *ND1*	1.137	3.118 (1.641 to 5.927)	0.001	2.151	8.592 (3.372 to 21.891)	<0.001
Saliva *ND4*	−0.252	0.777 (0.428 to 1.412)	0.408			
Saliva *ND5*	−0.704	0.495 (0.271 to 0.901)	0.022	−1.141	0.319 (0.114 to 0.891)	0.029
Saliva *ND6*	−0.925	0.397 (0.186 to 0.846)	0.017	−1.239	0.290 (0.100 to 0.838)	0.022
Saliva *CO1*	−0.537	0.584 (0.287 to 1.189)	0.138			
Saliva *CO2*	−0.501	0.606 (0.287 to 1.280)	0.189			
Saliva *CYTB*	−0.998	0.369 (0.178 to 0.763)	0.007	‒	‒	‒
PBMCs *IFIT1*	0.600	1.823 (1.287 to 2.581)	0.001	0.722	2.059 (1.272 to 3.331)	0.003
PBMCs *IFIT3*	0.666	1.946 (1.397 to 2.712)	<0.001	0.741	2.099 (1.394 to 3.160)	<0.001
PBMCs *IFI44*	0.026	1.027 (0.769 to 1.370)	0.858			
PBMCs *IFI44L*	0.277	1.319 (0.952 to 1.826)	0.096	‒	‒	‒
PBMCs *LY6E*	0.323	1.382 (0.946 to 2.019)	0.095	−0.715	0.489 (0.274 to 0.873)	0.016
PBMCs *OAS1*	−0.115	0.891 (0.681 to 1.167)	0.403			
PBMCs *MX1*	0.305	1.357 (0.992 to 1.854)	0.056	‒	‒	‒
PBMCs *ISG15*	−0.317	0.728 (0.474 to 1.119)	0.148			

* Variables with a p<0.1 in univariate models were included and selected using the forward selection method.

mt-RNAs, mitochondrial RNAs; PBMCs, peripheral blood mononuclear cells.

Based on the expression levels of ISGs measured in PBMCs, we calculated IFN scores commonly used in previous SjD-related studies. IFN score 1 was derived from *IFIT3, IFI44, IFI44L, LY6E* and *MX1*,^19^ while IFN score 2 was calculated using *IFIT1, IFIT3, IFI44, IFI44L* and *MX1*.^22^ When comparing to the previously reported IFN scores, our ISG score demonstrated the best performance for differentiating SjD from HC (online supplemental figure 2). Therefore, we used our proposed ISG score for subsequent analyses.

**Figure 2:**
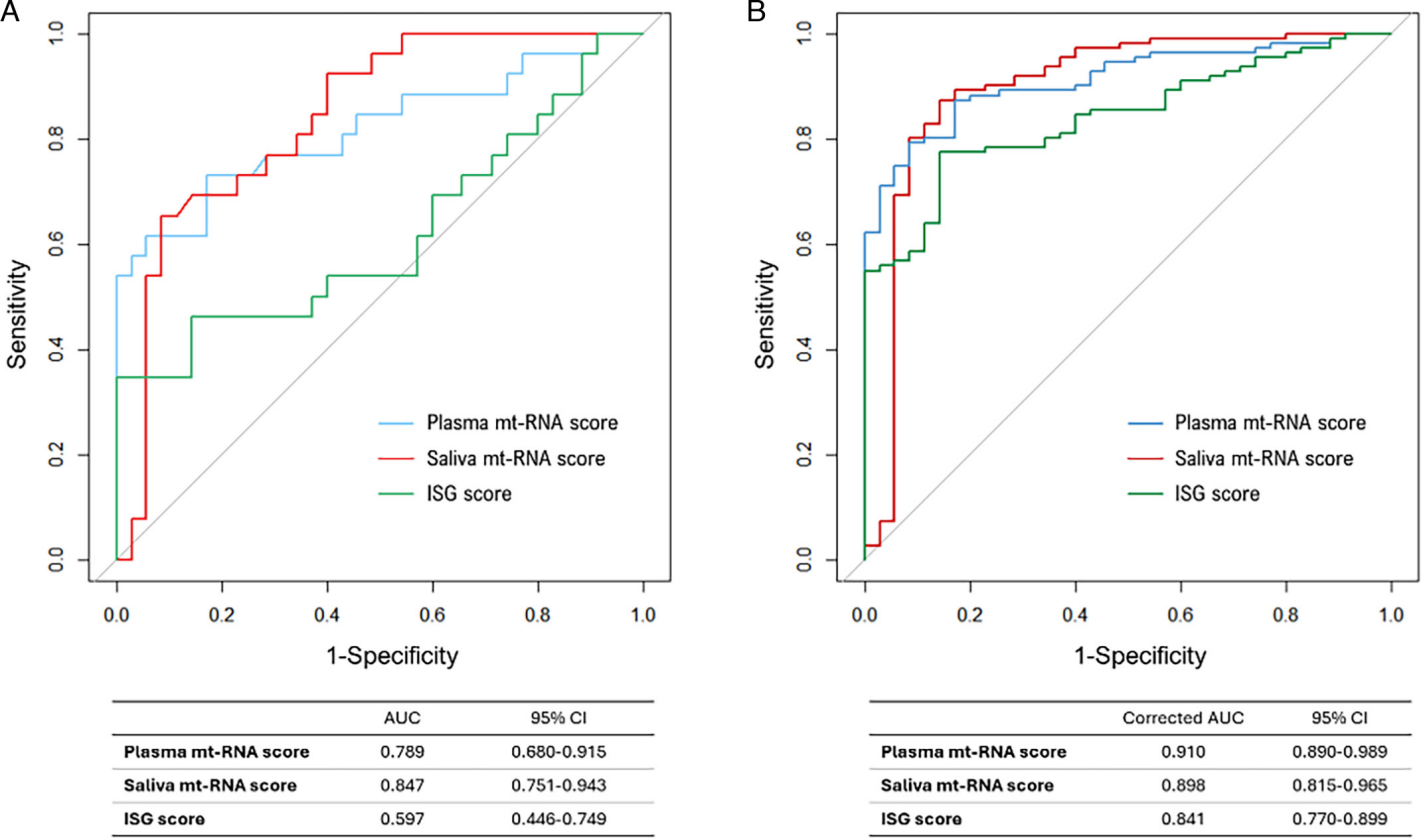
Receiver operating characteristics curves of each score for differentiating patients with Sjögren disease (SjD, n=26) and healthy controls (n=35) from the population with all biosamples (plasma, saliva, and PBMCs; (**A**) and the population with missing values imputed (**B**). AUC, area under the curve; ISG, interferon-stimulated gene; mt-RNA, mitochondrial RNA; PBMCs, peripheral blood mononuclear cells.

To evaluate the discriminative power of mt-RNA scores and our ISG score in differentiating SjD from HC, we performed ROC curve analysis (figure 2). Among participants with all three biosamples (plasma, saliva and PBMC), the AUC values of the plasma mt-RNA, saliva mt-RNA and ISG scores were 0.789, 0.847 and 0.597, respectively (figure 2A). The AUC values for the plasma and saliva mt-RNA scores were significantly higher than that for the PBMC ISG score (p=0.026 and 0.007, respectively, according to the DeLong test). After imputing missing values, we calculated the optimism-corrected AUCs for these scores. The corrected AUCs for plasma and saliva mt-RNA scores (0.910 and 0.898, respectively) were higher than that of ISG score (0.841), though without statistical significance (figure 2B).

### Comparison of plasma mt-RNA expression levels and mt-RNA scores among patients with SjD and the disease controls

**Figure 3:**
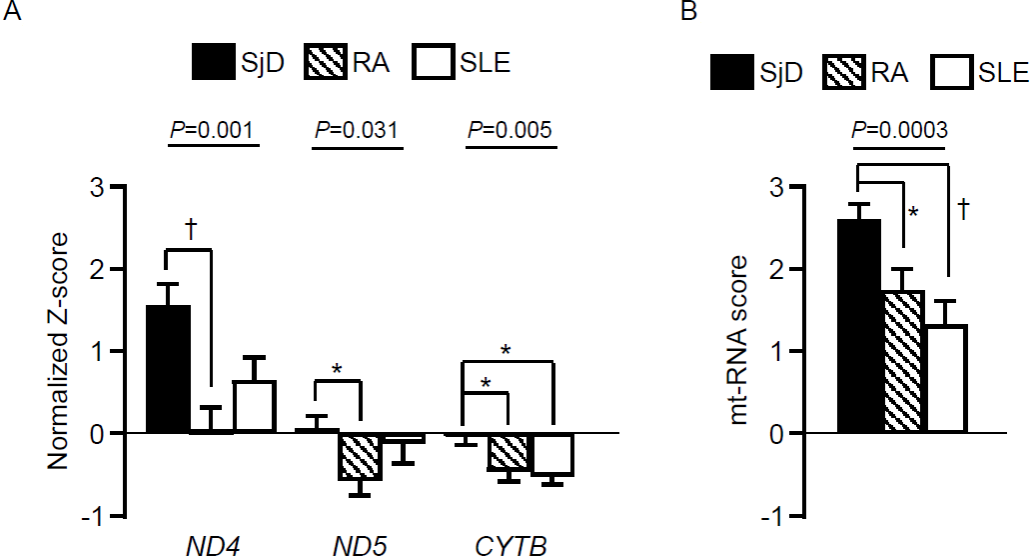
Comparisons of each plasma mitochondrial RNA (mt-RNA) expression levels (**A**) and plasma mt-RNA scores (**B**) among patients with Sjögren disease (SjD), rheumatoid arthritis (RA) and systemic lupus erythematosus (SLE). The p values in the upper panel were calculated using analysis of variance. *p<0.05; †p<0.01 when compared with SjD (Turkey post hoc test).

Given that ISGs are upregulated in other autoimmune diseases such as RA and SLE,^23 24^ we investigated mt-RNA expression levels and mt-RNA scores in plasma samples from patients with RA or SLE. The plasma levels of *ND4* (p=0.001) and *ND5* (p=0.023) were significantly higher in patients with SjD than in those with RA, whereas they were comparable between patients with SjD and SLE (figure 3). However, plasma *CYTB* levels and plasma mt-RNA scores were significantly higher in patients with SjD than in those with RA or SLE (both p<0.05, figure 3). Furthermore, the ROC curve analysis for plasma mt-RNA scores across the pooled data from the SjD, RA and SLE subgroups showed an AUC of 0.672 (95% CI 0.588 to 0.755, p<0.001, online supplemental figure 3), indicating a moderate ability to discriminate SjD. Considering that the proportion of patients taking immunoregulatory drugs, including glucocorticoids, was lower in the SjD subgroup than in the RA or SLE subgroups (online supplemental table 3), we conducted a multivariate regression analysis adjusting for the use of these drugs. We observed a significant association between plasma mt-RNA scores and SjD (OR=1.405, 95% CI 1.121 to 1.762, p=0.003).

### Associations between mt-RNA levels or scores and clinical features in patients with SjD

**Figure 4:**
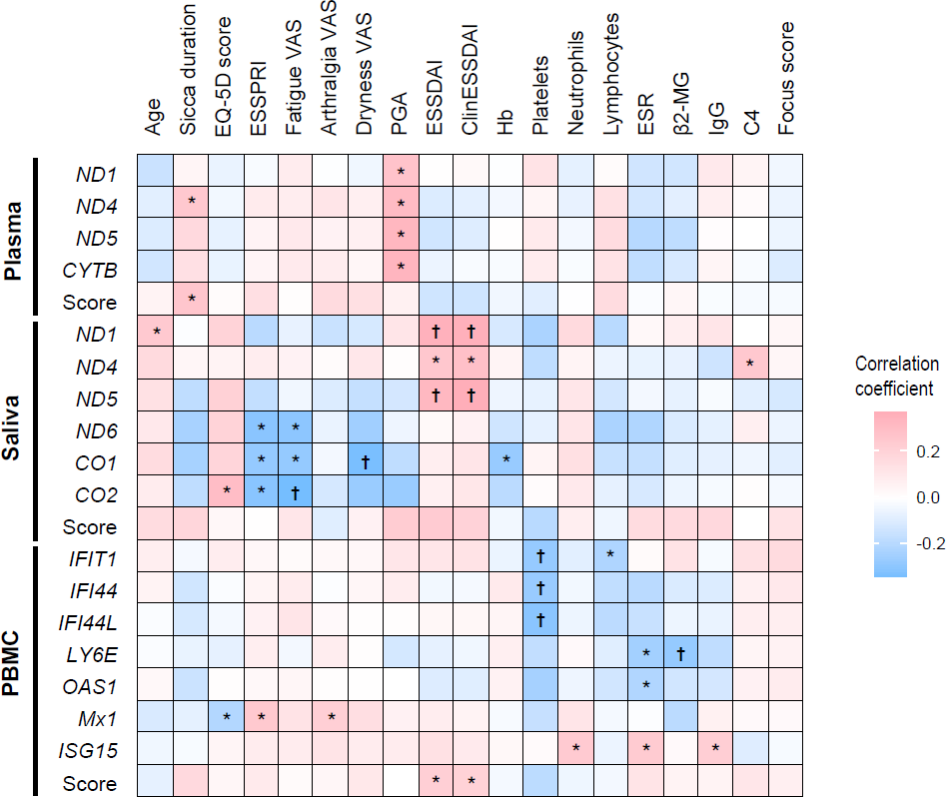
Heatmap showing correlations between individual mitochondrial RNA/interferon-stimulated gene (ISG) expressions or scores and the clinical features of Sjögren disease (SjD). *p<0.05, ^†^p<0.01. β2-MG, β2-microglobulin; ClinESSDAI, Clinical ESSDAI; C4, complement 4; ESR, erythrocyte sedimentation rate; ESSDAI, EULAR Sjögren Syndrome Disease Activity Index; ESSPRI, EULAR Sjögren Syndrome Patient Reported Index; EULAR, European Alliance of Associations for Rheumatology; Hb, haemoglobin; IgG, immunoglobulin G; PBMC, peripheral blood mononuclear cell; PGA, patients’ global assessment; EQ-5D, EuroQoL 5 Dimension; VAS, Visual Analogue Scale

To investigate the clinical implications of mt-RNA expression levels, we examined the correlations between individual mt-RNA expression levels or mt-RNA/ISG scores and the clinical variables of SjD (figure 4). Additionally, we compared mt-RNA levels or mt-RNA/ISG scores on the basis of the presence of SjD-related categorical variables (online supplemental table 4).

We found that several plasma mt-RNA expressions (*ND1*, *ND4*, *ND5* and *CYTB*) were positively associated with the patients’ global assessment (all p<0.05). *ND1*, *ND4*, and *ND5* mt-RNA levels in saliva and PBMC ISG scores were positively correlated with objective disease activity indices, such as ESSDAI and ClinESSDAI (all p<0.05). *MX1* expression in PBMCs was positively correlated with patient-reported ESSPRI and arthralgia Visual Analogue Scale (VAS), whereas salivary expression levels of *ND6*, *CO1* and *CO2* were negatively correlated with ESSPRI and fatigue VAS (all p<0.05; figure 4). The semiquantitative FS showed no significant correlation with any mt-RNA/ISG levels or scores. However, patients with SjD in the highest quartile of ESSDAI or ClinESSDAI (both p=0.032) and those with the Raynaud phenomenon (p=0.008) had significantly higher saliva mt-RNA scores than those without. PBMC ISG scores were also significantly higher in SjD patients with oral dryness, ClinESSDAI>5, or those in the highest quartile of ClinESSDAI (all p<0.05; online supplemental table 4).

In the disease control groups, plasma mt-RNA scores were significantly higher in RA patients with active disease (DAS28≥3.2) or anti-CCP negativity and those taking hydroxychloroquine than their counterparts (all p<0.01; online supplemental table 5). Among patients with SLE, plasma mt-RNA scores were significantly higher in those with moderate-to-high disease activity (SLEDAI≥6) or high disease activity (SLEDAI≥11) than their counterparts (all p<0.05; online supplemental table 5). Additionally, plasma mt-RNA scores showed a weak positive correlation with SLEDAI (*ρ*=0.325, p=0.041).

### Sensitivity analyses for mt-RNA scores in SjD subgroups

The ROC curve analysis was repeated after stratifying SjD patients based on the presence of anti-SSA/Ro, FS≥1 or serum IgG≥1.8 g/dL (online supplemental figures 4–6). ROC curve analyses demonstrated that plasma and saliva mt-RNA scores significantly distinguished all SjD subgroups from HCs. Additionally, the AUC value of the saliva mt-RNA score tended to be higher than that of PBMC ISG score in all SjD subgroups, although the difference was not statistically significant. Regarding the association between salivary mt-RNA scores and SjD disease activity, ESSDAI and ClinESSDAI showed significant correlations with saliva *ND1, ND4 or ND5* mt-RNA levels as well as the composite saliva mt-RNA score, although variations were observed across SjD subgroups. When evaluating the performance of plasma mt-RNA scores in discriminating SjD subgroups from autoimmune disease controls (SLE and RA), most analyses demonstrated fair to good discriminative ability.

## Discussion

This study demonstrated the differential expression of mt-RNAs in saliva and plasma, and ISGs in the PBMCs of patients with SjD. Composite mt-RNA scores derived from our results distinguished patients with SjD from HCs better than PBMC ISG scores. Additionally, plasma mt-RNA scores in patients with SjD were significantly higher than those in patients with RA and SLE. Moreover, salivary mt-RNA and PBMC ISG scores were related to higher ESSDAI and ClinESSDAI values in SjD, whereas plasma mt-RNA scores were associated with increased disease activity in patients with RA and SLE.

There is growing evidence that the cytosolic or extracellular release of mt-DNAs/RNAs as endogenous ligands activates type I IFN pathways, leading to autoimmune diseases.^7 25^ Additionally, mt-RNAs may act as autoantigens in patients with SLE.^26^ However, few studies have investigated the presence and importance of extracellular mt-RNAs in SjD. Our findings support the idea that mt-RNAs can serve as immunomodulatory factors in SjD, which is consistent with findings of our previous study.^8^ We previously demonstrated that cytoplasmic mt-RNA can activate PKR pathways and extracellular mt-RNA released from damaged cells can activate TLR3.^27^ We also observed elevated mt-RNA levels in the acinar regions of diseased NOD salivary glands. Suppressing mt-RNA transcription mitigated the detrimental effects of poly(I:C) and the protective effects of acetylcholine on ISG expression in salivary gland epithelial cells.^8^

In body fluids, mt-RNA may be free within exosomes or microparticles, or in cell-free mitochondria that are intact or damaged.^28 29^ Our study found decreased levels of plasma *ND1* and salivary *ND5*, *ND6* and *CYTB* but upregulated levels of plasma *ND4* and salivary *ND1* in patients with SjD. These mitochondrial genes are part of respiratory chain complexes. Moreover, patients with SjD showed higher plasma levels of *ND4*, *ND5* or *CYTB* than those with RA and SLE. Furthermore, mitochondrial dysfunction is associated with SjD, showing the downregulation of respiratory chain complex genes in SjD labial glands.^30 31^ Mitochondrial damage or dysfunction may release mt-RNAs into the cytoplasm and drive the senescence-associated secretory phenotypes observed in the immune cells of SjD labial glands.^32 33^

Despite the similar synthesis rates of mt-RNAs from the same mt-DNA strand, their steady states are influenced by multiple biological factors, including post-transcriptional modification, RNA stability and tissue-specific or cell-specific processing mechanisms.^34^ A human mitochondrial transcriptome analysis demonstrated unexpected complexities in the regulation, expression and processing of mt-RNA across multiple cell lines and tissues.^35^ The mechanisms underlying the differences in mt-RNA expression observed in our study remain unclear and warrant further investigation. Nevertheless, our findings suggest that extracellular mt-RNAs contribute to immune dysregulation and may serve as potential biomarkers for disease monitoring, although mt-RNA release may not be the sole driver of IFN activation in SjD.

In this study, plasma mt-RNA levels were not consistently associated with PBMC ISG levels. In contrast, some salivary mt-RNAs showed weak to moderate associations with PBMC ISG expression, indicating that these may reflect the primary impact of SjD on the exocrine glands. Furthermore, the weighted burden of extracellular mt-RNA (expressed as mt-RNA scores) in saliva and plasma significantly correlated with ISG scores (figure 1B). These findings suggest that the upregulated mt-RNA burden in biofluids may be mechanistically linked to ISG expression in patients with SjD, regardless of the specific mt-RNA transcript and independent of a direct association between mt-RNA score and mitochondrial dysfunction.

Composite scores from plasma and salivary mt-RNA levels provided significantly better discrimination between HCs and patients with SjD than the PBMC ISG scores (figure 2). Pooled analyses of patients with SjD, RA and SLE showed significantly higher plasma mt-RNA scores in SjD, with a moderate AUC value of 0.672 for distinguishing SjD from the autoimmune disease controls (online supplemental figure 3). Most patients with RA and SLE were taking anti-rheumatic drugs, but the significance of plasma mt-RNA scores remained even after adjusting for drug use. Since salivary mt-RNA or PBMC ISG scores were not available for our patients with RA and SLE, further validation is needed for plasma or salivary mt-RNA scores as SjD-specific diagnostic biomarkers.

Type I and/or II IFN pathways play a pathophysiological role in many rheumatic diseases by bridging innate and adaptive immune responses. However, no gold standard exists for assessing IFN signature, and the number and components of IFN-inducible genes and proteins differ largely across studies.^36^ A recent systematic review revealed that IFN pathway activation correlates with disease activity in SLE, RA and SjD, although most studies have a high or unclear risk of bias.^36^ Among the four qRT-PCR-based studies evaluating multiple ISGs (*IFI44*, *IFI44L*, *IFIT1*, *IFIT3* and *MX1*) in whole blood or monocytes of SjD,^19 37^ two found an association between the type I IFN signature and ESSDAI, whereas the other two found no significant relationship.^38 39^ These studies calculated an ‘IFN score’ as a non-weighted sum of standardised scores for five ISGs. In contrast, our ISG score was derived by weighing the standardised scores of differentially expressed genes among eight PBMC ISGs, demonstrating superior discriminative performance to previously defined methods (online supplemental figure 2). Furthermore, PBMC ISG scores were significantly associated with objective SjD disease activity indices (ESSDAI and ClinESSDAI). Additionally, patients with SjD in the highest quartile of the ESSDAI or ClinESSDAI exhibited higher salivary mt-RNA scores, which were significantly correlated with PBMC ISG scores (online supplemental table 4). These findings highlight the potential of our weighted ISG score and mt-RNA burden as biomarkers for disease activity in SjD.

Meanwhile, PBMC *MX1* expression in our study showed a positive correlation with ESSPRI, which contrasts with previous reports that found no significant association between the IFN signature and ESSPRI or even lower IFN scores in fatigued SjD patients.^40 41^ However, a recombinant nuclease therapy demonstrated significant improvement in fatigue and ESSPRI levels in patients with SjD and mental fatigue scores were correlated with the expression of ISGs including *MX1* in responders.^42^ Additionally, IL-36α is overexpressed in the salivary gland and circulation of SjD patients and has been reported to be more upregulated in fatigued patients with SjD.^41^ IL-36α could amplify type I IFN signalling including *MX1*.^43^ These findings may explain the association observed in our study.

In contrast to salivary mt-RNA scores, plasma mt-RNA scores were not associated with any clinical variables in our patients with SjD, although the IFN-inducing capacity of SjD sera was reportedly related to dermatologic, haematologic, and pulmonary manifestations.^44^ However, patients with active disease (DAS28≥3.2 for RA or SLEDAI≥6 for SLE) showed higher plasma mt-RNA scores than those with inactive RA and SLE. Significant associations between mt-DNA levels and disease activity in SLE, RA, and anti-neutrophil cytoplasmic antibody-associated vasculitis have also been reported.^45^,^47^ This discrepancy may be associated with a low systemic inflammatory burden in patients with SjD. Collectively, these findings suggest that circulating mitochondrial NAs contribute to the pathogenesis of systemic rheumatic diseases, or that their disease activity is associated with mitochondrial dysfunction.

In our study, salivary mt-RNA scores were significantly higher in patients with Raynaud phenomenon than in those without. Raynaud phenomenon is the most frequently reported non-ESSDAI feature in patients with SjD and is known adverse reaction of systemic IFN therapy.^48 49^ Moreover, PBMC ISG scores were elevated in patients with oral dryness, which is a leading symptom of SjD. Apart from these associations, no specific clinical features including FS in the MSG and anti-SSA/Ro positivity were linked to mt-RNA scores. Thus, from the perspective of extracellular RNA in the pathogenesis of SjD, the mitochondrial NA burden might affect the overall disease status but not specific organ involvement.

The mitochondrial genome is bidirectionally transcribed to generate long complementary RNAs, which can spontaneously form dsRNAs. Because of the biochemical stability of the dsRNA structure, mt-RNAs can be detected in body fluid samples, unlike mRNA strands derived from genomic DNAs.^28 50^ This structural stability suggests that mt-RNAs may serve as more reliable biomarkers than ISG mRNA for assessing SjD-related changes in biofluid samples. However, the relative contribution of mt-RNAs to the IFN signature remains unknown in SjD. Notably, treatment with the recombinant RNase (RSLV-132) improved fatigue scores in patients with SjD, with the improvements correlating with elevated PBMC ISG expression at baseline.^42^ If mt-RNAs play a substantial role in SjD pathogenesis, the therapeutic efficacy of RNase-based agents may be limited, as the dsRNA structure of mt-RNAs confers resistance to RNase-mediated degradation.

Our study has several limitations. First, the sample size was relatively small. Second, not all patients provided paired saliva, plasma and PBMC samples, limiting comprehensive analysis. UWS samples were not available from patients with severe salivary dysfunction. Third, as a cross-sectional study, it cannot determine whether altered mt-RNA levels drive or results from SjD, necessitating longitudinal studies. Fourth, while we performed internal validation using sensitivity analyses, external validation in an independent SjD cohort is required. Fifth, our data-driven mt-RNA and ISG score were designed to reduce individual variation, affected by unknown mechanisms, and optimise disease association. Although our ISG score outperformed previous IFN signature scores, further validation is needed. Lastly, the main cellular sources of mt-RNA in saliva and plasma remain unclear. Poor correlations in mt-RNA expression levels among samples may reflect diverse mt-RNA-releasing cells. Thus, our findings do not confirm a specific mt-RNA’s role in the IFN pathway or validate mt-RNA scores as an index of mitochondrial dysfunction. Despite these limitations, this is the first to investigate mt-RNA burdens in saliva and plasma and their association with clinical features and PBMC ISG expression levels, providing novel insights into SjD pathogenesis.

In conclusion, our results suggest that the extracellular mt-RNA burden is higher in patients with SjD than in HCs and that mt-RNA scores can distinguish patients with SjD from HCs and those with RA or SLE. Moreover, salivary mt-RNA scores were associated with objective SjD disease activity indices. Our results highlight the potential role of extracellular mt-RNA in the pathogenesis of SjD, indicating that further research is needed to establish it as a biomarker for disease monitoring and stratification of SjD.

## Supplementary material

10.1136/rmdopen-2024-005166 online supplemental file 1

## Data Availability

All data relevant to the study are included in the article or uploaded as supplementary information.
