# Supplementary material for: Association of mitochondrial RNA expression levels in saliva and plasma with interferon signature gene expression and disease activity in patients with Sjögren disease
Source: RMD Open. 2025 May 13;11(2):e005166. doi: 10.1136/rmdopen-2024-005166 (PMC12083325; doi:10.1136/rmdopen-2024-005166)
Supplement: online supplemental file 1 [file rmdopen-11-2-s001.pdf]

Supplementary Table S1 Basic demographic information of study participants and number of collected samples

	Healthy donors (n = 35)	SjD (n = 111)	RA (n = 40)	SLE (n = 40)	*P*-value
Age (years)	47.7 ± 10.2	47.8 ± 11.8	51.5 ± 12.7	46.4 ± 12.0	0.302*
Women	35 (100)	106 (95.5)	37 (92.5)	40 (100)	0.292†
Body mass index (kg/m^2)	22.4 ± 2.9	22.2 ± 2.9	22.8 ± 4.3	22.7 ± 4.7	0.805*
	(n = 17)	(n = 95)	(n = 37)		
Disease activity at the time of sampling	–	ESSDAI 3.7 ±3.1	DAS28 5.03 ± 1.40	SLEDAI 3.9 ± 4.4	
Active disease		ESSDAI ≥5: 25 (22.5)	DAS 28 ≥3.2: 34 (85.0)	SLEDAI >4: 29 (72.5)	
Number of collected samples					
Unstimulated whole saliva	35 (100)	65 (56.8)	–	–	
Plasma	35 (100)	79 (71.2)	40 (100)	40 (100)	
PBMCs	35 (100)	103 (92.8)	–	–	

Values are presented as mean ± standard deviation or number (%).

SjD, Sjögren disease; RA, rheumatoid arthritis; SLE, systemic lupus erythematosus; ESSDAI, EULAR Sjögren's Syndrome Disease Activity, Index; DAS28, Disease Activity Score 28; SLEDAI, Systemic Lupus Erythematosus Disease Activity Index; PBMCs, peripheral blood mononuclear cells.

*, p-values using ANOVA; †, p-values using chi-square test.

Supplementary Table S2 Primer sequences for RT-qPCR

Gene	Forward Primer (5'-3')	Reverse Primer (3'-5')
CO1 (heavy strand)	GCCATAACCCAATACCAAACG	CGCAAATGGGCGGTAGGCGTG
CO2 (heavy strand)	CTAGTCCTGTATGCCCTTTTCC	CGCAAATGGGCGGTAGGCGTG
CYTB (heavy strand)	CAATTATACCCTAGCCAACCCC	CGCAAATGGGCGGTAGGCGTG
ND1 (light strand)	GTTGTGATAAGGGTGGAGAGG	CGCAAATGGGCGGTAGGCGTG
ND4 (heavy strand)	CTCACACTCATTCTCAACCCC	CGCAAATGGGCGGTAGGCGTG
ND5 (heavy strand)	CTAGGCCTTCTTACGAGCC	CGCAAATGGGCGGTAGGCGTG
ND6 (heavy strand)	TCATACTCTTTCACCCACAGC	CGCAAATGGGCGGTAGGCGTG
IFIT1	CAGAATGAGGAAGCCCTGAA	CGCAAATGGGCGGTAGGCGTG
IFIT3	GTTCTCTTGGGCCTGAAACT	CGCAAATGGGCGGTAGGCGTG
IFI44	GGTCATTGAGCTCAGGAAGAG	CGCAAATGGGCGGTAGGCGTG
IFI44L	CAGGACTGTGCATGGATGA	CGCAAATGGGCGGTAGGCGTG
LY6E	TCCGACCAGGACAACTACT	CGCAAATGGGCGGTAGGCGTG
OAS1	TGTGTGTCCAAGGTGGTAAAG	CGCAAATGGGCGGTAGGCGTG
MX1	CTGCATCCCACCCTCTATTAC	CGCAAATGGGCGGTAGGCGTG
ISG15	CTCATCTTTGCCAGTACAGGAG	CGCAAATGGGCGGTAGGCGTG
GAPDH	CTCTCTGCTCCTCCTGTTCGAC	CGCAAATGGGCGGTAGGCGTG

Supplementary Table S3 Drugs prescribed within 3 months prior to enrollment

Drugs	SjD (n=111)	SLE (n=40)	RA (n=40)
Pilocarpine	19 (17.1)	1 (2.5)	0 (0.0)
NSAIDs	14 (12.6)	4 (10.0)	23 (57.5)
Glucocorticoids	9 (8.1)	25 (62.5)	14 (35.0)
PD equivalent dose (mg/day)	3.8 ± 2.3	10.9 ± 19.7	4.0 ± 1.4
Hydroxychloroquine (HCQ)	22 (19.8)	31 (77.5)	5 (12.5)
HCQ dose (mg/day)	284 ± 81	265 ± 61	260 ± 55
Other anti-rheumatic drugs			
Methotrexate	1 (0.9)	5 (12.5)	15 (37.5)
Sulfasalazine	1 (0.9)	0 (0.0)	2 (5.0)
Bucillamine	1 (0.9)	0 (0.0)	1 (2.5)
Mycophenolate	1 (0.9)	8 (20.0)	0 (0.0)
Azathioprine	1 (0.9)	6 (15.0)	0 (0.0)
Leflunomide	0 (0.0)	0 (0.0)	1 (2.5)
Cyclosporine A	0 (0.0)	3 (7.5)	0 (0.0)
Tacrolimus	0 (0.0)	7 (17.5)	1 (2.5)
Cyclophosphamide	0 (0.0)	2 (5.0)	0 (0.0)
Belimumab	0 (0.0)	1 (2.5)	0 (0.0)

Values are presented as mean ± standard deviation or number (%).

SjD, Sjögren disease; RA, rheumatoid arthritis; SLE, systemic lupus erythematosus; NSAIDs, non-steroidal anti-inflammatory drugs; PD, prednisolone.

Supplementary Table S4 Comparisons of mt-RNA or interferon-stimulated genes (ISG) scores according to clinical features of Sjögren disease

	Clinical variable	Yes	No	*p*-value
Saliva mt-RNA score	Highest quartile of ESSDAI or ClinESSDAI	3.298 ± 1.506 (n=16)	2.386 ± 1.424 (n=49)	0.032
	Raynaud phenomenon	3.819 ± 1.893 (n=9)	2.416 ± 1.331 (n=56)	0.008
PBMC ISG score	Oral dryness	2.582 ± 1.775 (n=65)	1.787 ± 2.042 (n=38)	0.041
	ClinESSDAI >5	3.305 ± 2.093 (n=24)	2.087 ± 1.815 (n=79)	0.015
	Highest quartile of ClinESSDAI	3.139 ± 2.207 (n=17)	2.030 ± 1.741 (n=86)	0.012

ISG, IFN-stimulated gene; ESSDAI, EULAR Sjögren's Syndrome Disease Activity; ClinESSDAI, Clinical ESSDAI; PBMC, peripheral blood mononuclear cell.

Supplementary Table S5 Comparisons of plasma mt-RNA score according to clinical features of RA or SLE

	Clinical variable	Yes	No	*p*-value
RA	Active disease (DAS28 ≥3.2)	2.021 ± 1.145 (n=34)	0.144 ± 1.764 (n=6)	0.007
	Anti-CCP positivity	1.663 ± 1.670 (n=37)	2.677 ± 0.154 (n=3)	0.001
	Use of hydroxychloroquine	2.646 ± 0.347 (n=5)	1.610 ± 1.700 (n=35)	0.003
SLE	Moderate-to-high disease activity (SLEDAI ≥6)	2.719 ± 1.708 (n=8)	0.966 ± 1.717 (n=32)	0.014
	High disease activity (SLEDAI ≥11)	3.330 ± 0.890 (n=3)	1.153 ± 1.801 (n=37)	0.047

RA, rheumatoid arthritis; SLE, systemic lupus erythematosus; DAS28, Disease Activity Score 28; anti-CCP, anti-cyclic citrullinated peptide; SLEDAI, Systemic Lupus Erythematosus Disease Activity Index.

Supplementary Figure S1 Correlations among the expression of plasma mt-RNA, saliva mt-RNA, and PBMC IFN-stimulated genes in patients with Sjögren disease. *p<0.05, †p<0.01, ‡p<0.001.

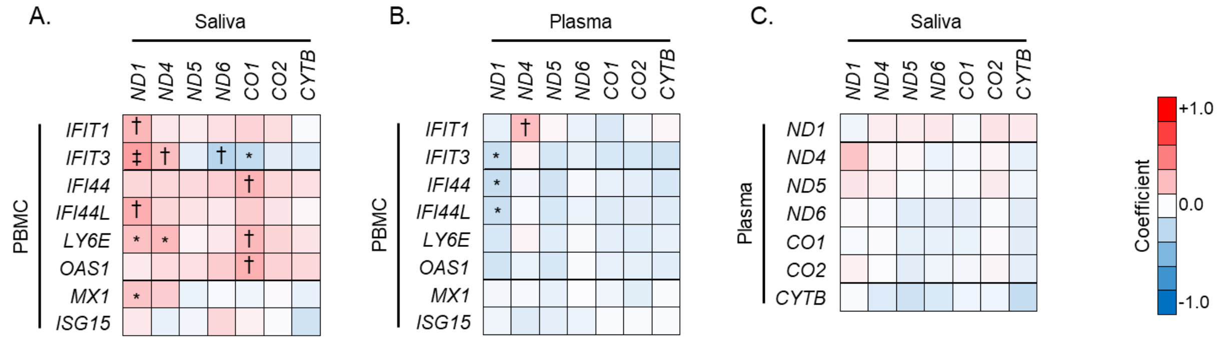

Supplementary Figure S2 Receiver operating characteristic curves of our interferon-stimulated gene (ISG) score and previously suggested interferon (IFN) scores to differentiate patients with Sjögren disease and healthy controls.

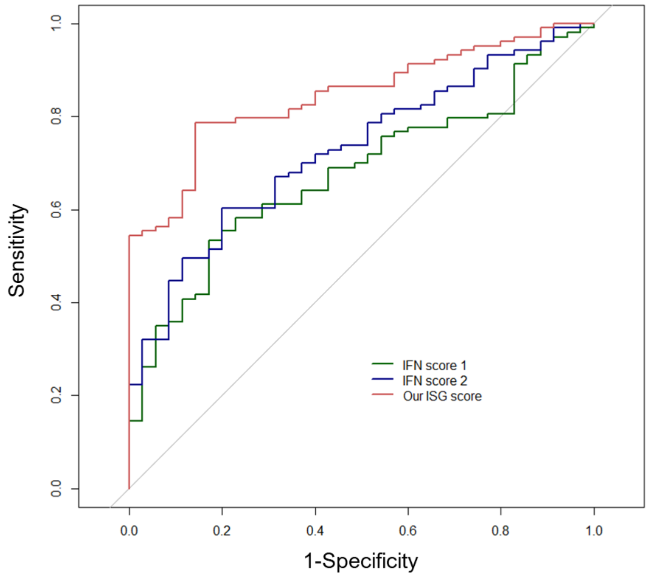

	AUC	Standard error	95% CI	P-value
**Our ISG score**	0.844	0.033	0.780-0.909	<0.001
**IFN score 1**†	0.677	0.047	0.584-0.771	0.002
**IFN score 2**‡	0.726	0.045	0.637-0.815	<0.001

† *IFIT3, IFI44, IFI44L, LY6E, MX1*
‡ *IFIT1, IFIT3, IFI44, IFI44L, MX1*

Supplementary Figure S3 The receiver operating characteristics curve of plasma mt-RNA score to differentiate Sjögren disease (SjD) among pooled data from SjD, rheumatoid arthritis, and systemic lupus erythematosus.

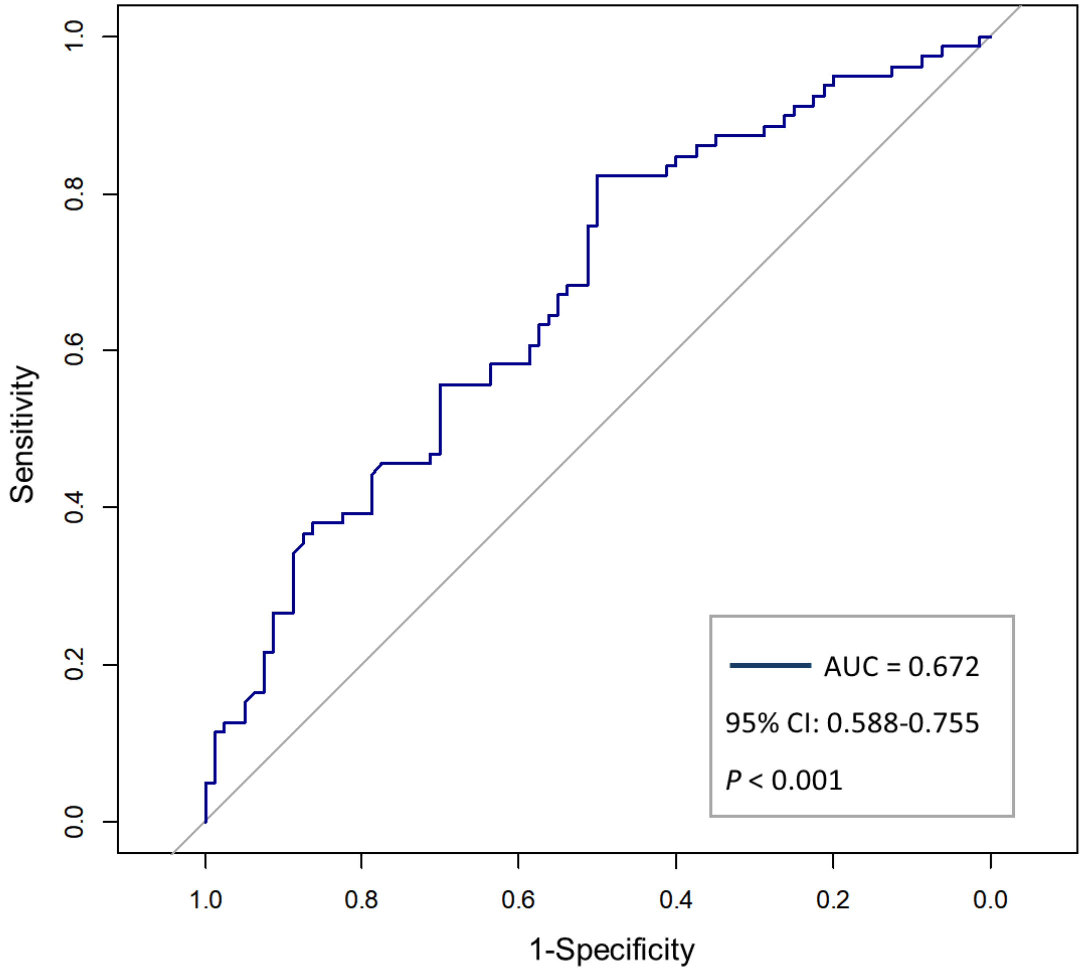

Supplementary Figure S4 Sensitivity analysis including only patients with Sjögren disease (SjD) who were anti-SSA/Ro positive (n=101) or negative (n=10). (A) Receiver operating characteristics (ROC) curve for distinguishing patients with SjD from healthy controls. (B) Pearson correlation coefficients between SjD disease activity and saliva levels of *ND1*, *ND4*, or *ND5* mitochondrial (mt)-RNA and mt-RNA scores. (C) ROC curve for distinguishing patients with SjD from those with rheumatoid arthritis and systemic lupus erythematosus.

(A)

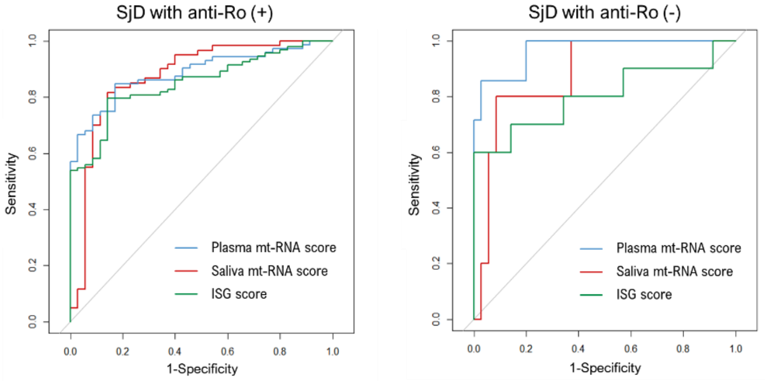

	AUC	95% CI
**Plasma mt-RNA score**	0.885	0.824-0.947
**Saliva mt-RNA score**	0.872	0.790-0.955
**ISG score**	0.849	0.783-0.915

	AUC	95% CI
**Plasma mt-RNA score**	0.967	0.907-1.000
**Saliva mt-RNA score**	0.880	0.735-1.000
**ISG score**	0.803	0.602-1.000

(B)

	**ESSDAI**	**ClinESSDAI**
Anti-Ro (+) SjD		
ND1	0.365†	0.375†
ND4	0.243	0.285*
ND5	0.390†	0.435†
mt-RNA score	0.229	0.202
Anti-Ro (-) SjD		
ND1	0.383	0.163
ND4	0.630	0.562
ND5	-0.366	-0.420
mt-RNA score	0.700	0.472

*$P < 0.05$, †$P < 0.01$.

(C)

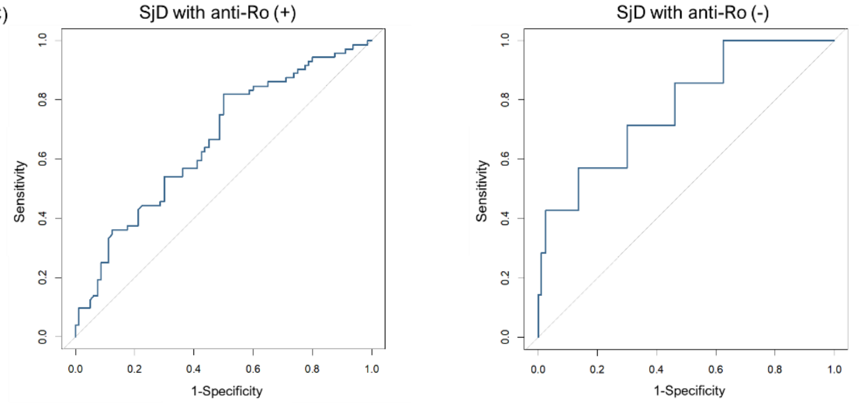

AUC	Standard error	95% CI	*P*-value
0.661	0.044	0.575-0.748	< 0.001

AUC	Standard error	95% CI	*P*-value
0.777	0.097	0.588-0.966	0.004

Supplementary Figure S5 Sensitivity analysis including only patients with Sjögren disease (SjD) who had focus score ≥1 (n=77) or <1 (n=24). (A) Receiver operating characteristics (ROC) curve for distinguishing patients with SjD from healthy controls. (B) Pearson correlation coefficients between SjD disease activity and saliva levels of *ND1*, *ND4*, or *ND5* mitochondrial (mt)-RNA and mt-RNA scores. (C) ROC curve for distinguishing patients with SjD from those with rheumatoid arthritis and systemic lupus erythematosus.

(A)

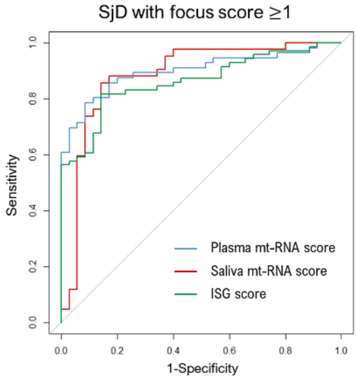

	AUC	95% CI
**Plasma mt-RNA score**	0.899	0.835-0.963
**Saliva mt-RNA score**	0.884	0.799-0.968
**ISG score**	0.862	0.794-0.931

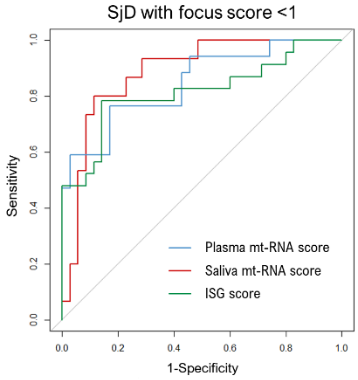

	AUC	95% CI
**Plasma mt-RNA score**	0.845	0.726-0.964
**Saliva mt-RNA score**	0.886	0.790-0.982
**ISG score**	0.815	0.691-0.939

(B)

	**ESSDAI**	**ClinESSDAI**
SjD with FS ≥1		
ND1	0.402†	0.410†
ND4	0.351*	0.419†
ND5	0.020	0.132
mt-RNA score	0.441†	0.371*
SjD with FS <1		
ND1	0.455	0.466
ND4	0.420	0.445
ND5	0.732†	0.692†
mt-RNA score	0.229	0.255

**P* < 0.05, †*P* < 0.01.

(C)

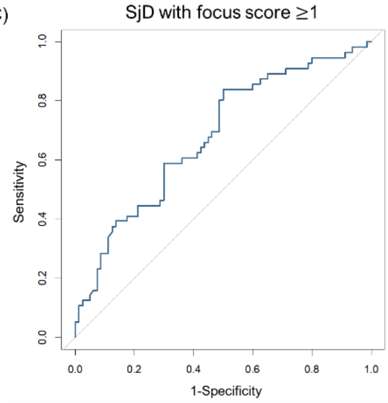

AUC	Standard error	95% CI	*P*-value
0.680	0.047	0.589-0.771	<0.001

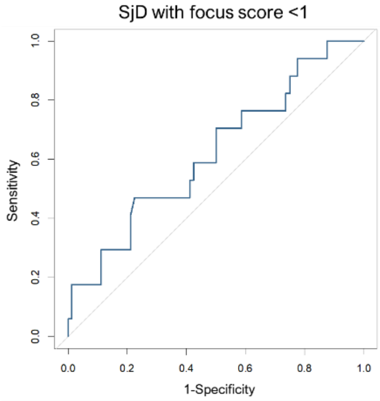

AUC	Standard error	95% CI	*P*-value
0.620	0.077	0.468-0.772	0.121

Supplementary Figure S6 Sensitivity analysis including only patients with Sjögren disease (SjD) who had serum total IgG ≥1.8 g/dL (n=74) or <1.8 g/dL (n=37). (A) Receiver operating characteristics (ROC) curve for distinguishing patients with SjD from healthy controls. (B) Pearson correlation coefficients between SjD disease activity and saliva levels of *ND1*, *ND4*, or *ND5* mitochondrial (mt)-RNA and mt-RNA scores. (C) ROC curve for distinguishing patients with SjD from those with rheumatoid arthritis and systemic lupus erythematosus.

(A)

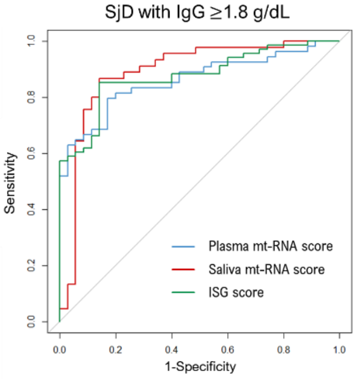

	AUC	95% CI
**Plasma mt-RNA score**	0.860	0.785-0.936
**Saliva mt-RNA score**	0.888	0.806-0.971
**ISG score**	0.875	0.809-0.941

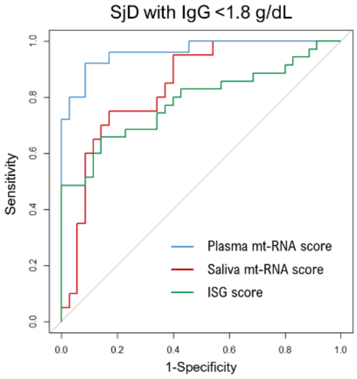

	AUC	95% CI
**Plasma mt-RNA score**	0.962	0.919-1.000
**Saliva mt-RNA score**	0.839	0.732-0.945
**ISG score**	0.785	0.675-0.896

(B)

	ESSDAI	ClinESSDAI
SjD with IgG ≥ 1.8 g/dL		
ND1	0.328*	0.352*
ND4	0.295*	0.309*
ND5	0.432†	0.453†
mt-RNA score	0.155	0.155
SjD with IgG < 1.8 g/dL		
ND1	0.438	0.428
ND4	0.420	0.426
ND5	0.058	0.088
mt-RNA score	0.450*	0.408

**P* < 0.05, †*P* < 0.01.

(C)

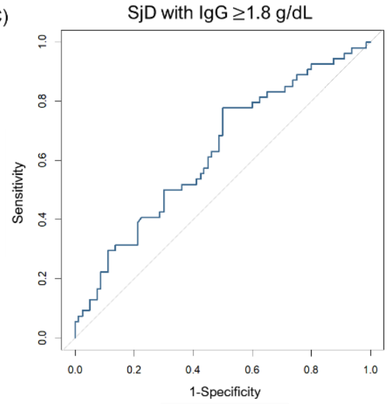

AUC	Standard error	95% CI	*P*-value
0.629	0.049	0.533-0.725	0.009

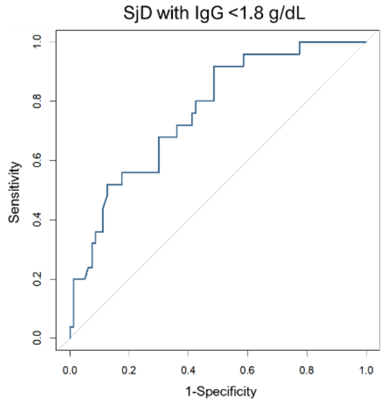

AUC	Standard error	95% CI	*P*-value
0.763	0.052	0.662-0.865	<0.001
